# Hyperprogression of a mismatch repair-deficient colon cancer in a humanized mouse model following administration of immune checkpoint inhibitor pembrolizumab

**DOI:** 10.18632/oncotarget.28086

**Published:** 2021-10-12

**Authors:** Ilyas Sahin, Andrew George, Shengliang Zhang, Kelsey E. Huntington, Zehra Ordulu, Lanlan Zhou, Wafik S. El-Deiry

**Affiliations:** ^1^Laboratory of Translational Oncology and Experimental Cancer Therapeutics, The Warren Alpert Medical School of Brown University, Providence, RI, USA; ^2^Joint Program in Cancer Biology, Brown University and Lifespan Health System, Providence, RI, USA; ^3^Division of Hematology/Oncology, The Warren Alpert Medical School of Brown University, Providence, RI, USA; ^4^Cancer Center at Brown University, The Warren Alpert Medical School of Brown University, Providence, RI, USA; ^5^Department of Pathology & Laboratory Medicine, The Warren Alpert Medical School of Brown University, Providence, RI, USA; ^6^Pathobiology Graduate Program, Warren Alpert Medical School of Brown University, Providence, RI, USA; ^7^Department of Pathology, Massachusetts General Hospital, Harvard Medical School, Boston, MA, USA; ^8^Molecular and Cellular Biology Graduate Program, The Warren Alpert Medical School of Brown University, Providence, RI, USA; ^9^Present Address: University of Florida Health Cancer Center, Gainesville, FL, USA; ^*^These authors contributed equally to this work

**Keywords:** cancer immunotherapy, immune checkpoint inhibitors, hyperprogressive disease (HPD), hyperprogression (HP), humanized mouse

## Abstract

Immunotherapy is an established treatment modality in oncology. However, in addition to primary or acquired therapy resistance with immune checkpoint blockade (ICB), hyperprogressive disease (HPD) or hyperprogression (HP) with acceleration of tumor growth occurs in a subset of patients receiving ICB therapy. A validated and predictive animal model would help investigate HPD/HP to develop new approaches for this challenging clinical entity. Using human cytotoxic T-cell line TALL-104 injected intraperitoneally into immunodeficient NCRU-nude athymic mice bearing mismatch repair-deficient (MMR-d) human colon carcinoma HCT116 p53-null (but not wild-type p53) tumor xenograft, we observed accelerated tumor growth after PD-1 blockade with pembrolizumab administration. There was increased colon tumor cell proliferation as determined by immunohistochemical Ki67 staining of tumor sections. There was no increase in MDM2 or MDM4/MDMX in the p53-null HCT116 cells versus the wild-type p53-expressing isogenic tumor cells, suggesting the effects in this model may be MDM2 or MDM4/MDMX-independent. Human cytokine profiling revealed changes in IFN-γ, TRAIL-R2/TNFRSF10B, TRANCE/TNFSF11/RANK L, CCL2/JE/MCP-1, Chitinase 3-like 1, IL-4 and TNF-α. This represents a novel humanized HPD mouse model with a link to deficiency of the p53 pathway of tumor suppression in the setting of MMR-d. Our novel humanized preclinical TALL-104/p53-null HCT116 mouse model implicates p53-deficiency in an MMR-d tumor as a possible contributor to HPD/HP and may help with evaluating therapeutic strategies in cancer immunotherapy to extend clinical benefits of ICB’s in a broader patient population.

## INTRODUCTION

Immune checkpoint inhibitors (ICI’s) have revolutionized cancer treatment with significant responses, and an explosion of new single or combination therapies involving the immune system have been approved by the FDA for multiple cancers. However, efficacy of ICI’s is limited due to primary or secondary resistance. Furthermore, among the non-responders to ICI’s, the phenomenon called hyperprogressive disease (HPD) or hyperprogression (HP) with acceleration of tumor growth is observed in patients (5–29%) with a dramatic progression of disease [1, 2]. Genomic alterations such as MDM2 or MDM4/MDMX amplification, EGFR alterations and several genes located on chromosome 11q13 have been reported to be linked to HPD [3–5].

Although there are early insights, the molecular and cellular mechanisms underlying HPD/HP, and predictive markers have not been established [2, 6]. Currently, one of the major obstacles in understanding the pathophysiology of HPD/HP is a lack of preclinical experimental *in vivo* models, making it difficult to study this phenomenon.

We present a humanized mouse model recapitulating HPD/HP in cancer immunotherapy which might help with understanding HPD/HP biology and with identification of possible new treatments for this clinical situation which has very limited treatment options.

## RESULTS

### Accelerated tumor growth in a novel humanized mouse model after anti-PD-1 therapy, pembrolizumab

**Figure 1:**
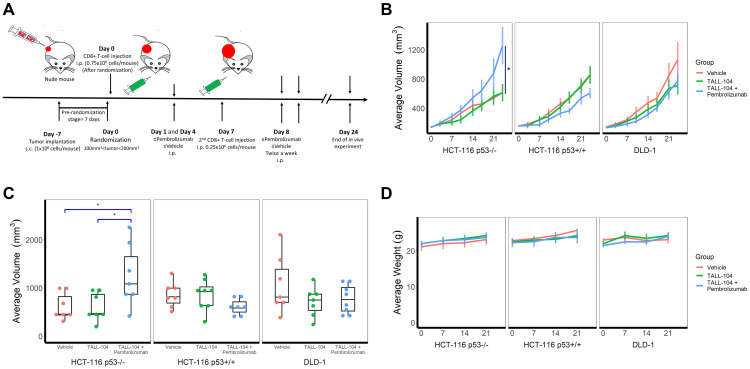
Humanized hyperprogressive disease (HPD)/hyperprogression (HP) cancer immunotherapy model. (**A**) Experimental design schema to humanize NCRU nude athymic mice with human TALL-104 CD8+ cytotoxic T-cell line. Mice (*n* = 7 for all groups except HCT-116 p53+/+ plus TALL-104 and DLD-1 plus TALL-104 with Pembrolizumab for which *n* = 8) were given subcutaneous injections of human colorectal carcinoma cell lines to induce tumor formation before a humanized T-cell system was established through intraperitoneal injection of TALL-104 cells in suspension and mice were treated with anti-PD-1 (pembrolizumab) or vehicle control (PBS). (**B**) Growth curves for human CRC xenograft cell lines, measured by caliper twice weekly. Line shows mean group tumor volume ± 1 SEM. (**C**) Distribution of tumor volumes for individual mice within each treatment group at end of the experiment (day 24). (**D**) Mouse body weights as measured throughout the experiment. Line shows mean group weight in grams ± 1 SEM.

**Figure 2:**
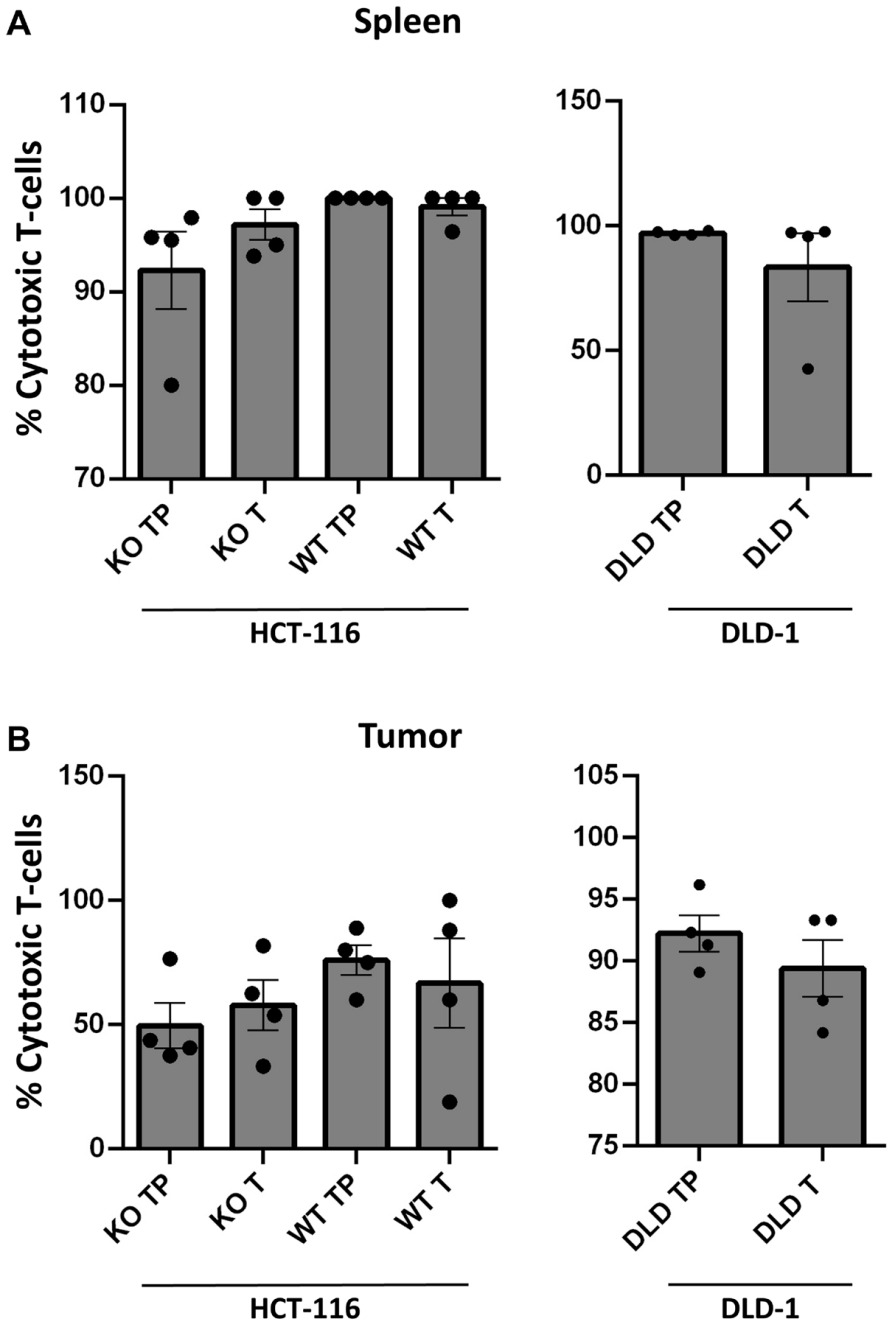
Establishment of TALL-104 in circulation within humanized mouse models. (**A**–**B**) Flow cytometric analysis from blood of humanized mice across treatment groups (*n* = 4 per group). Gating strategy of live/CD45+/CD3+/CD4- used to identify TALL-104 CD8+ cytotoxic T-cells in humanized athymic mice. All samples obtained upon sacrifice at end of experiment. p53−/− is abbreviated as KO, p53+/+ is abbreviated as WT, T represents groups which received TALL-104 i.p. injection, and TP represents groups which recieved TALL-104 i.p. injection as well as anti-PD-1 therapy. (A) Flow cytometric analysis of overall circulation for CD8+ cells from spleen of humanized mice. (B) Flow cytometric analysis of tumor environment for CD8+ cells from tumor xenograft of humanized mice.

Using athymic mice, we generated xenograft models of subcutaneous human CRC tumors of cell lines HCT-116 p53−/−, HCT-116 p53+/+, and DLD-1 (mutant p53/Ser^241^Phe). We, then, performed intraperitoneal (i.p.) injection of TALL-104, CD8+ human Cytotoxic T cells (CTLs), before treating with anti-PD-1 therapy (pembrolizumab) i.p. twice weekly for 24 days to determine the effects of the therapy on tumor growth (Figure 1A). Mean tumor volume of each group (*n* = 7 or 8), measured twice weekly through caliper measurements, revealed a significantly elevated growth in humanized mice receiving anti-PD-1 immunotherapy unique to HCT-116 p53−/− xenografts (*P* = 0.04 relative to both non-humanized and humanized controls, Figure 1B blue line). In an independently performed repeat *in vivo* experiment using HCT-116 p53−/− xenografts with TALL-104 ± pembrolizumab (*n* = 4 for each group), we observed similar results confirming the HPD phenotype (Supplementary Figure 1). In contrast, the same therapy demonstrated a downward trend in tumor growth for HCT-116 p53+/+ xenografts, though this difference was not significant by the time mice were required to be sacrificed due to institutional policy and ethical concerns (Figure 1B, second graph blue line). Non-humanized mice (lacking TALL-104) bearing HCT-116 xenografts (both p53−/− and p53+/+) showed identical trends to humanized mice receiving no treatment in terms of tumor growth (Figure 1B first two graphs, red line). We also conducted the same experiment on a third human CRC cell line, DLD-1, for which the presence of T-cells alone in the humanized model showed a downward trend in tumor growth relative to the non-humanized mice lacking T-cells, with no effect of anti-PD-1 therapy, though once again this trend was not significant by the time mice were required to be sacrificed (Figure 1B third graph). The distribution of tumor volumes for individual mice within each treatment group at the end of the study is also shown (Figure 1C). Finally, we monitored mouse weight and condition throughout the experiment to assess any potential toxicity either from the establishment of the humanized model or anti-PD-1 therapy and observed no difference in overall condition or weight between all groups (HCT-116 p53−/−, *P* = 0.72; HCT-116 p53+/+, *P* = 0.33; DLD-1, *P* = 0.55, Figure 1D). Establishment and activity of injected T-cells in the humanized model were assessed in the periphery through flow cytometry analysis of the spleen (Figure 2A) and in the tumor environment through flow cytometry analysis of a section of the tumor (Figure 2B), both upon sacrifice at end of experiment.

**Figure 3:**
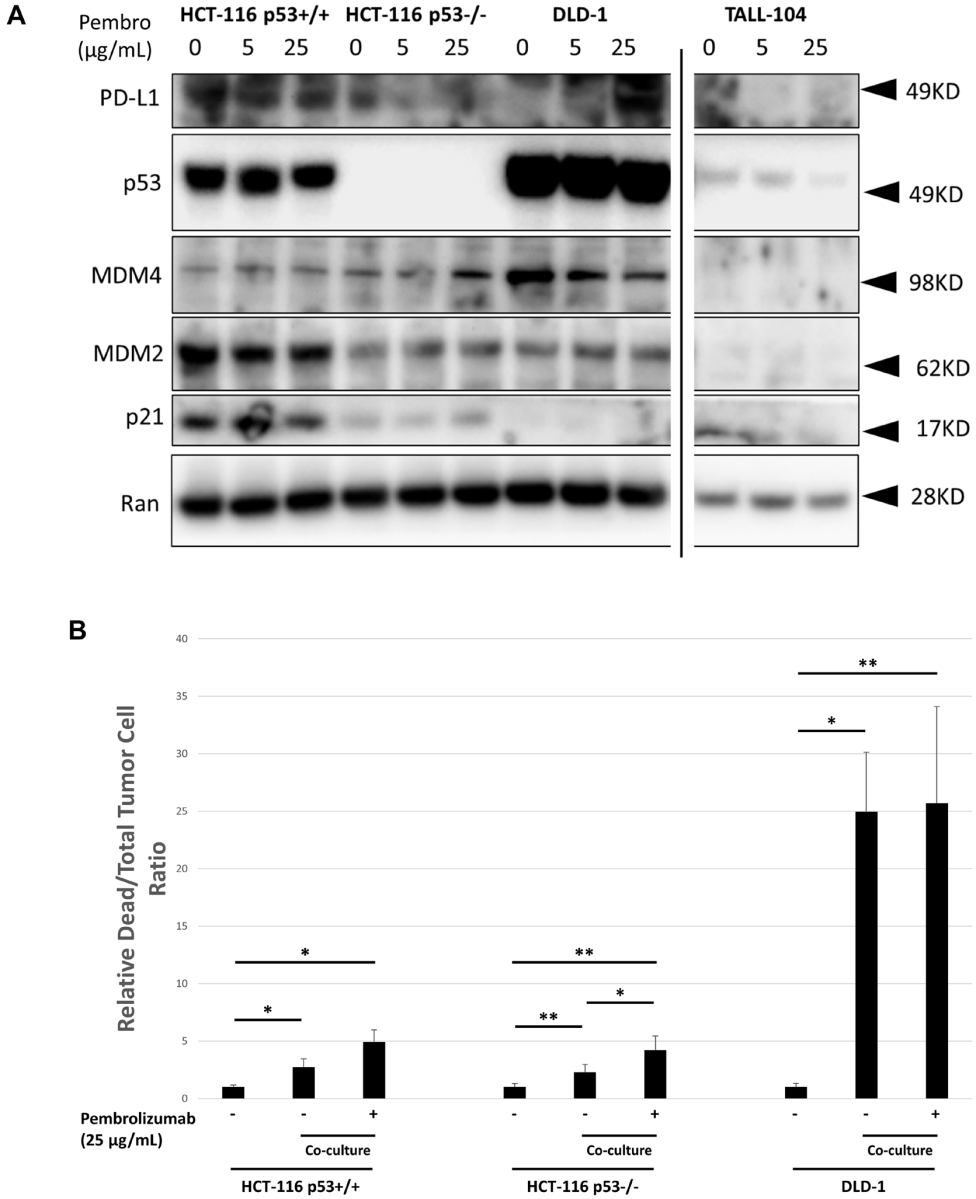
*In vitro* characterization of human CRC cell line response to co-culture with TALL-104 human CD8+ T-cells and anti-PD-1 therapy. (**A**) Western blot showing expression levels of key proteins following 20 h different dose levels of pembrolizumab treatment for CRC cell lines or TALL-104 CD8+ human T-cell line. Ran was used for loading control. (**B**) 24 h co-culture experiment where human CRC cell lines were cultured with TALL-104 CD8+ human T-cell line and treated with pembrolizumab or control to measure T-cell killing *in vitro*.

We simultaneously examined expression levels of PD-L1, p53 and related proteins in all cell lines in response to different dose levels of pembrolizumab using western blotting (Figure 3A). We performed *in vitro* T-cell co-culture experiments of CRC cell lines with and without pembrolizumab (Figure 3B). HCT-116 cell lines were resistant to T-cell mediated killing but showed increased T-cell mediated killing in co-culture in the presence of pembrolizumab. DLD-1, on the other hand, was more sensitive to T-cell mediated killing but no effect was observed in the presence of pembrolizumab. Cell viability through CTG for co-cultures was also assessed for all cell lines, but there was no evidence suggesting an accelerated growth rate within a 5-day co-culture period (Data not shown).

### Enhanced Ki-67 expression in xenograft tumor of the HPD mouse model

**Figure 4:**
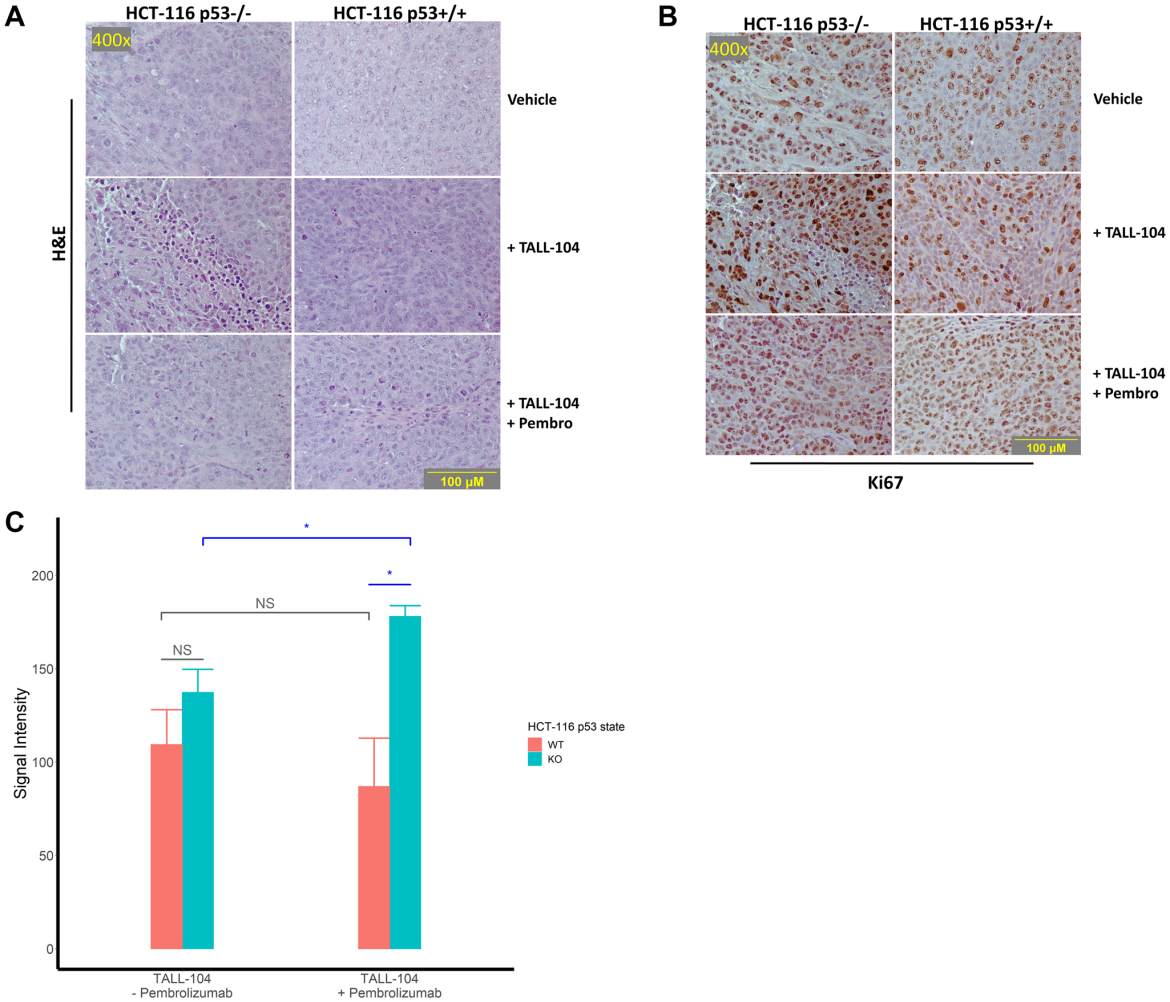
Histological assessment of tumor sections for proliferation. (**A**–**B**) Representative images of stained sections from excised and formalin-fixed tumors at the end of the experiment. All images were captured at 400× magnification and corresponding images are taken from corresponding fields on different sections. (A) Representative H&E tumor histology at 400× magnification. (B) Representative immunohistochemical staining against the proliferation marker Ki67 imaged at 400× magnification. (**C**) Quantification of staining intensity of proliferation marker Ki67 in humanized mice. For analysis, 3 fields were imaged from 3 tumors per humanized group and analyzed. “NS” means not significant.

We maintained the treatment schedule and continued to monitor tumor growth until mouse tumor volume surpassed the ethical limit (2000 mm^3^ or approximately 10% of body weight), at which point mice were sacrificed and a portion of resected HCT-116 p53−/− and HCT-116 p53+/+ tumors was fixed in formalin and embedded in paraffin. Then, we first surveyed xenograft histology through a haematoxylin and eosin (H&E) stain, finding clear establishment of injected CRC xenograft with minimal T-cell infiltration (Figure 4A). Next, we stained sections of the same tissue for proliferation marker Ki67 (Figure 4B). Quantification of Ki67 stain in non-necrotic tissue lacking T-cell infiltration for humanized mice revealed a significant increase in expression for HCT-116 p53−/− xenograft mice upon anti-PD-1 therapy (*P* = 0.03; Figure 4C). Direct comparison between humanized mice receiving anti-PD-1 therapy also showed a significantly higher expression level for HCT-116 p53−/− xenograft mice compared with HCT-116 p53+/+ xenograft mice (*P* = 0.03; Figure 4C).

### Human cytokine profiling of the HPD model

**Figure 5:**
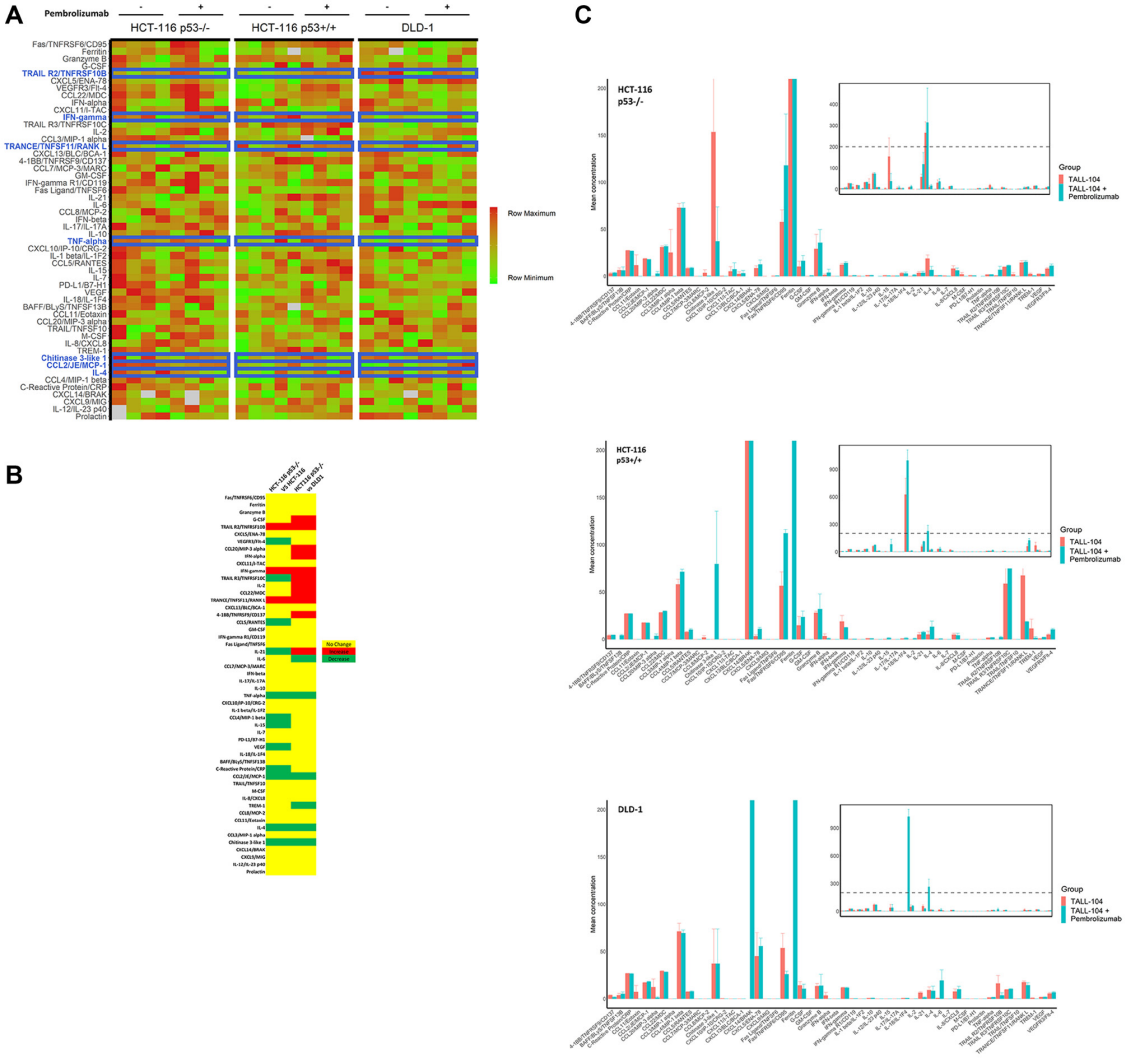
Cytokine profiling of peripheral blood in mice. Purified plasma collected from humanized mice upon sacrifice at end of experiment was analyzed with a broad-spectrum human cytokine panel (*n* = 4 per humanized mouse for all groups except HCT-116 p53+/+ without anti-PD-1 for which *n* = 5). (**A**) Heat map of relative cytokine expression levels for individual mice. Row maximum is shown in full red and row minimum is shown in full green across all cell lines. Cytokines are ranked in decreasing order of absolute treatment effect of cytokine expression for HCT-116 p53−/−, such that the top represents cytokines for which anti-PD-1 treatment yielded the most increase in expression and the bottom represents cytokines for which anti-PD-1 treatment yielded the most decrease in expression. Cytokines and corresponding rows highlighted in blue represent key cytokines for which a significant treatment effect was observed for HCT-116 p53−/− that differed from the treatment effect for both HCT-116 p53+/+ and DLD-1. NA values are shown in grey. (**B**) Heat map showing significant differences in treatment effects between HCT-116 p53−/− and other cell lines. Yellow represents no significant difference in treatment effect between HCT-116 p53−/− and cell line being compared, while red represents a significant increase in treatment effect in HCT-116 p53−/− compared with other cell line and green represents a significant decrease in treatment effect in HCT-116 p53−/− compared with other cell line. Rows for which the same effect is observed when comparing HCT-116 p53−/− to both HCT-116 p53+/+ and DLD-1 correspond to rows highlighted in blue in A. (**C**) Quantified mean cytokine levels ± 1 SEM (in pg/mL) for humanized mice receiving vehicle or anti-PD-1 therapy. Inset shows same data on broader y-axis scale.

**Figure 6:**
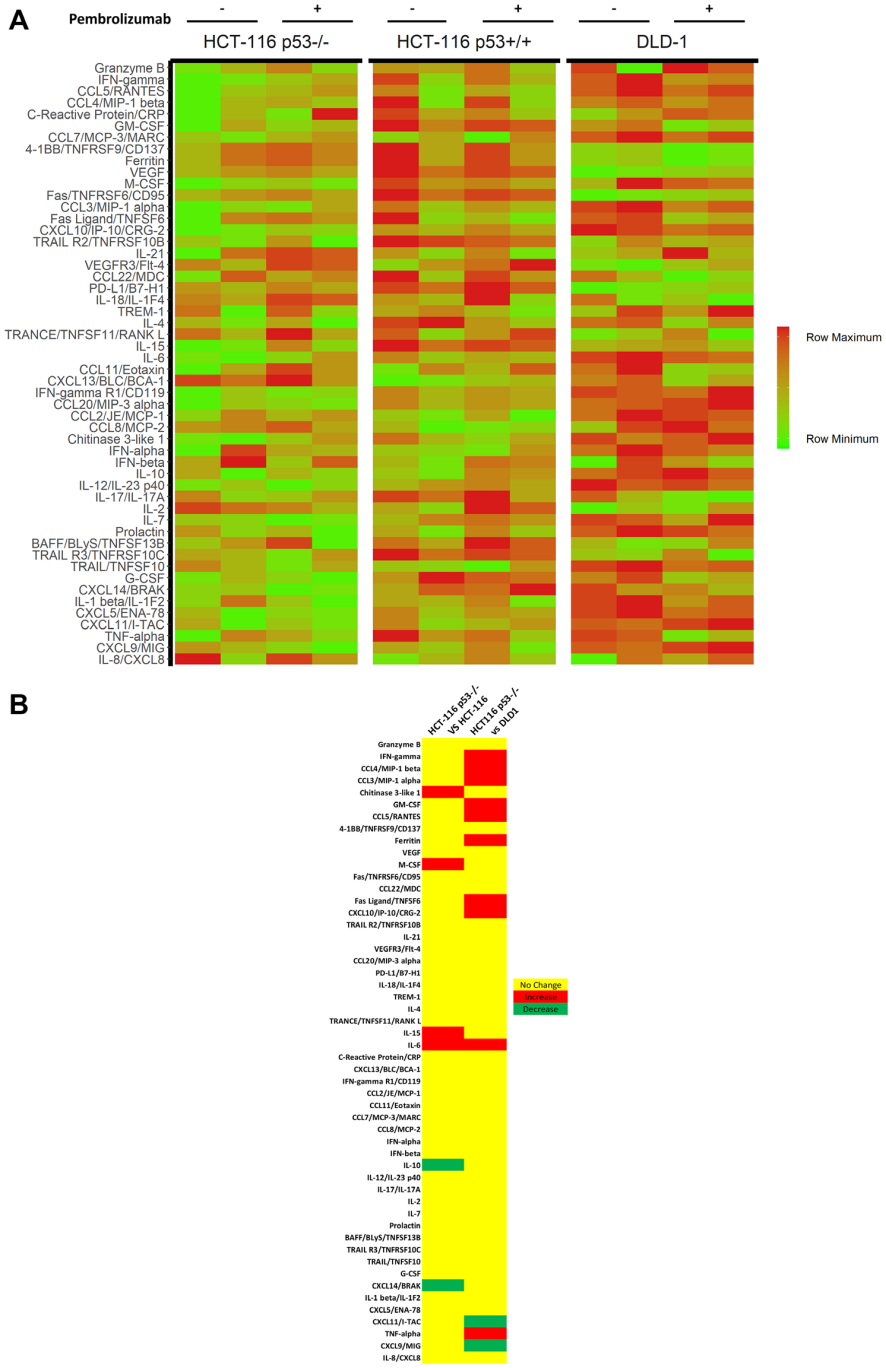
Cytokine profiling of *in vitro* CRC cell lines and TALL-104 co-cultures. Cytokine analysis was conducted on co-culture media following 24 hours of co-culture between CRC cell line and TALL-104, with and without 25 μg/mL pembrolizumab treatment. All experiments were conducted in quadruplets. (**A**) Heat map of cytokine expression levels in culture media following 24 h co-culture. (**B**) Significant cytokine differences in treatment response upon administration of anti-PD-1 therapy between cell lines.

Upon sacrifice, we collected blood from humanized mice (*n* = 5 untreated vs 4 treated for HCT-116 p53+/+; *n* = 4 untreated vs 4 treated for HCT-116 p53−/− and DLD-1) and analyzed the purified plasma for a panel of human cytokines (Figure 5A–5C). Overall results were largely heterogenous, though a comparison of treatment effect, defined as mean difference in cytokine expression under anti-PD-1 therapy, between HCT-116 p53−/− xenograft mice and others revealed a unique pattern in 15 cytokines relative to HCT-116 p53+/+ xenografts (increased in HCT-116 p53−/− relative to HCT-116 p53+/+: TRANCE, *P* = 0.004; IFN-γ, *P* = 0.003; TRAIL R2, *P* = 0.02; decreased in HCT-116 p53−/− relative to HCT-116 p53+/+: TNF-α, *P* = 0.008; CCL2, *P* = 0.0005; CRP, *P* = 0.001; IL-15, *P* = 0.00005; VEGF, *P* = 0.0000003; CCL5, 0.002; VEGFR3, *P* = 0.03; IL-21, *P* = 0.04; CCL4, *P* = 0.000007; TRAIL R3, *P* = 0.0004; Chitinase 3-like 1, *P* = 0.0004) and 18 cytokines relative to DLD-1 xenografts (increased in HCT-116 p53−/− relative to DLD-1: Fas, *P* = 0.003; TRAIL R2, *P* = 0.0003; CCL20, *P* = 0.002; G-CSF, *P* = 0.02; IFN-α, *P* = 0.006; IL-21, *P* = 0.0001; TRANCE, *P* = 0.02; 4-1BB, *P* = 0.00008; IFN-γ, *P* = 0.02; CCL20, *P* = 0.03; IL-2, *P* = 0.002; TRAIL R3, *P* = 0.04; decreased in HCT-116 p53−/− relative to DLD-1: TNF-α, *P* = 0.02; TREM-1, *P* = 0.007; CCL2, *P* = 0.00001; IL-4, *P* = 0.002; IL-6, *P* = 0.003; Chitinase 3-like 1, *P* = 0.01) (Tables 1 and 2, Figure 5B). Seven cytokines (IFN-γ, TRAIL R2, TRANCE, CCL2, Chitinase 3-like 1, IL-4, and TNF-α) were identified to have fully unique trends in HCT-116 p53−/− compared to both HCT-116 p53+/+ and DLD-1 (Table 3, Figure 5A blue highlights). We conducted a similar cytokine profile on *in vitro* samples (4 independent replicates) obtained from co-culture of CRC cell lines with TALL-104 and administration of anti-PD-1 therapy to see if the same patterns emerged, but fewer cytokines of relevance were found, and results were overall different (Figure 6A). Only one cytokine (IL-6) was identified *in vitro* to have a unique response to treatment for HCT-116 p53−/− relative to HCT-116 p53+/+ and DLD-1 (Figure 6B). Significant cytokine changes *in vitro* between groups are shown in detail (Tables 4 and 5).

**Table 1:** Significant differences in treatment effect on cytokines between HCT-116 p53−/− and HCT-116 p53+/+ *in vivo*

Cytokine	Change (HCT p53−/− vs HCT p53+/+)	HCT-116 p53−/−			HCT-116 p53+/+			Absolute difference of treatment effect (pg/mL)	*P*
		Absolute difference after treatment (pg/mL)	SD (pg/mL)	*n*	Absolute difference after treatment (pg/mL)	SD (pg/mL)	*n*		
TRANCE/TNFSF11/RANK L	Increased	0.75	2.61	8	−48.81	37.69	9	49.56	4.2 e-3
IFN-γ	Increased	1.99	2.05	8	−6.68	6.21	9	8.67	2.8 e-3
TRAIL R2/TNFRSF10B	Increased	6.14	3.78	8	2.12	0.96	9	4.02	2.0 e-2
TNF-α	Decreased	−0.02	0.24	8	0.36	0.27	9	−0.38	7.6 e-3
CCL2/JE/MCP-1	Decreased	−0.71	0.29	8	−0.11	0.26	9	−0.60	4.6 e-4
C-Reactive Protein/CRP	Decreased	−0.53	0.34	8	0.09	0.29	9	−0.62	1.1 e-3
IL-15	Decreased	−0.19	0.36	8	1.11	0.56	9	−1.30	5.0 e-5
VEGF	Decreased	−0.27	0.40	8	1.39	0.37	9	−1.66	3.1 e-7
CCL5/RANTES	Decreased	0.35	1.03	8	2.52	1.27	9	−2.17	1.5 e-3
VEGFR3/Flt-4	Decreased	2.94	2.39	8	5.35	1.25	9	−2.41	2.8 e-2
IL-21	Decreased	0.09	2.18	8	2.50	2.34	9	−2.41	4.4 e-2
CCL4/MIP-1 β	Decreased	−0.11	7.07	8	12.94	6.15	9	−13.06	1.2 e-3
IL-4	Decreased	−12.28	5.29	8	8.12	7.10	9	−20.40	7.4 e-6
TRAIL R3/TNFRSF10C	Decreased	1.60	0.73	8	66.24	32.85	9	−64.64	3.6 e-4
Chitinase 3-like 1	Decreased	−116.41	95.89	8	79.48	55.99	9	−195.89	3.6 e-4

Statistical analysis was carried out on mean effect of anti-PD-1 therapy on cytokine expression between mice with HCT-116 p53−/− and with HCT-116 p53+/+ xenografts (*n **=*** 4 untreated and 4 treated for HCT-116 p53−/− and 5 untreated and 4 treated for HCT-116 p53+/+) to reveal 15 cytokines with significant differences in expression levels following treatment between xenograft cell lines.

**Table 2:** Significant differences in treatment effect on cytokines between HCT-116 p53−/− and DLD-1 *in vivo*

Cytokine	Change (HCT p53−/− vs DLD-1)	HCT-116 p53−/−			DLD-1			Absolute difference of treatment effect (pg/mL)	*P*
		Absolute difference after treatment (pg/mL)	SD (pg/mL)	*n*	Absolute difference after treatment (pg/mL)	SD (pg/mL)	*n*		
Fas/TNFRSF6/CD95	Increased	56.05	56.05	8	−27.42	15.96	8	87.69	2.7 e-3
TRAIL R2/TNFRSF10B	Increased	3.78	3.78	8	−12.57	8.82	8	18.71	3.1 e-4
CCL20/MIP-3 α	Increased	2.18	2.18	8	−11.10	8.54	8	13.74	2.3 e-3
G-CSF	Increased	8.23	8.23	8	−3.71	6.31	8	9.96	1.8 e-2
IL-21	Increased	2.18	2.18	8	−5.07	1.17	8	5.16	1.1 e-4
TRANCE/TNFSF11/RANK L	Increased	2.61	2.61	8	−3.10	2.92	8	3.85	1.5 e-2
4-1BB/TNFRSF9/CD137	Increased	1.08	1.08	8	−2.06	0.64	8	2.68	7.6 e-5
IFN-γ	Increased	2.05	2.05	8	−0.19	0.68	8	2.18	2.0 e-2
CCL22/MDC	Increased	1.88	1.88	8	−1.04	0.51	8	1.83	2.9 e-2
IL-2	Increased	0.60	0.60	8	0.02	0.01	8	1.04	1.9 e-3
TRAIL R3/TNFRSF10C	Increased	0.73	0.73	8	0.79	0.70	8	0.81	4.1 e-2
TNF-α	Decreased	0.24	0.24	8	0.26	0.19	8	−0.28	2.3 e-2
TREM-1	Decreased	1.05	1.05	8	−0.62	0.36	8	−1.37	7.3 e-3
CCL2/JE/MCP-1	Decreased	0.29	0.29	8	1.09	0.57	8	−1.81	9.8 e-6
CCL8/MCP-2	Decreased	3.34	3.34	8	0.00	0.00	8	−3.18	3.1 e-2
IL-4	Decreased	5.29	5.29	8	−0.84	6.28	8	−11.43	1.6 e-3
IL-6	Decreased	0.20	0.20	8	18.57	11.39	8	−18.55	2.5 e-3
CCL3/MIP-1 α	Decreased	24.77	24.77	8	0.00	0.00	8	−24.77	2.5 e-2
Chitinase 3-like 1	Decreased	95.89	95.89	8	0.00	52.17	8	−116.41	1.2 e-2

Statistical analysis was carried out on mean effect of anti-PD-1 therapy on cytokine expression between mice with HCT-116 p53−/− and DLD-1 xenografts (*n **=*** 4 untreated and 4 treated for both HCT-116 p53−/− and DLD-1) to reveal 18 cytokines with significant differences in expression levels following treatment between xenograft cell lines.

**Table 3:** Cytokines with unique treatment effect in HCT-116 p53−/− xenografts as compared to other human CRC xenografts

Cytokine	Treatment Effect (pg/mL)^1^			Relative difference for HCT-116 p53−/−
	DLD-1	HCT-116 p53+/+	HCT-116 p53−/−	
IFN-γ	−0.19 ± 0.24	−6.68 ± 2.20	1.99 ± 0.72	Increased
TRAIL R2/TNFRSF10B	−12.57 ± 3.12	2.12 ± 0.34	6.14 ± 1.34	Increased
TRANCE/TNFSF11/RANK L	−3.10 ± 1.03	−48.81 ± 13.33	0.75 ± 0.92	Increased
CCL2/JE/MCP-1	1.09 ± 0.20	−0.11 ± 0.09	−0.71 ± 0.12	Decreased
Chitinase 3-like 1	0.00 ± 18.44	79.48 ± 19.80	−116.41 ± 33.90	Decreased
IL-4	−0.84 ± 2.22	8.12 ± 2.51	−12.28 ± 1.87	Decreased
TNF-α	0.26 ± 0.07	0.36 ± 0.10	−0.02 ± 0.08	Decreased

^1^Treatment effect shown as difference of mean cytokine expression following treatment plus or minus one standard error of the mean. Common patterns in cytokine expression level changes following treatment revealed 7 cytokines with unique response in HCT-116 p53−/− not observed in other tested cell lines.

**Table 4:** Significant differences in treatment effect on cytokines between HCT-116 p53−/− and HCT-116 p53+/+ *in vitro*

Cytokine	Change (HCT p53−/− vs HCT p53+/+)	HCT-116 p53−/−			HCT-116 p53+/+			Absolute difference of treatment effect (pg/mL)	*P*
		Absolute difference after treatment (pg/mL)	SD (pg/mL)	*n*	Absolute difference after treatment (pg/mL)	SD (pg/mL)	*n*		
Chitinase 3-like 1	Increased	174.90	89.38	4	−68.41	129.40	4	243.31	0.0250
M-CSF	Increased	72.62	66.42	4	−95.85	95.19	4	168.47	0.0310
IL-6	Increased	0.31	0.14	4	−0.13	0.28	4	0.43	0.0450
IL-15	Increased	0.31	0.22	4	−0.06	0.14	4	0.37	0.0370
IL-10	Decreased	0.00	0.09	4	0.23	0.07	4	−0.23	0.0091
CXCL14/BRAK	Decreased	−2.30	0.00	4	6.44	4.55	4	−8.74	0.0310

Cytokine profiling conducted on *in vitro* co-cultures (4 independent replicates) between human CRC cell lines HCT-116 p53−/− and HCT-116 p53+/+ and TALL-104 revealed 6 cytokines with significantly different treatment effects following 24 h anti-PD-1 treatment (25 μg/mL pembrolizumab).

**Table 5:** Significant differences in treatment effect on cytokines between HCT-116 p53−/− and DLD-1 *in vitro*

Cytokine	Change (HCT p53−/− vs DLD-1)	HCT-116 p53−/−			DLD-1			Absolute difference of treatment effect (pg/mL)	*P*
		Absolute difference after treatment (pg/mL)	SD (pg/mL)	*n*	Absolute difference after treatment (pg/mL)	SD (pg/mL)	*n*		
IFN-γ	Increased	416.25	197.00	4	−524.39	79.64	4	940.64	0.00095
TNF-α	Increased	−9.55	254.35	4	−696.99	60.07	4	687.44	0.01000
CCL4/MIP-1 β	Increased	334.01	339.58	4	−263.86	197.92	4	597.87	0.03000
GM-CSF	Increased	159.99	224.34	4	−355.26	112.55	4	515.25	0.01200
CCL3/MIP-1 α	Increased	193.25	152.27	4	−203.80	131.38	4	397.05	0.00790
CCL5/RANTES	Increased	110.89	84.82	4	−65.72	76.25	4	176.61	0.02200
Ferritin	Increased	86.57	77.49	4	−51.69	7.63	4	138.26	0.03700
Fas Ligand/TNFSF6	Increased	−7.74	20.35	4	−30.74	7.07	4	48.48	0.01300
IL-6	Increased	0.31	0.14	4	−38.07	16.34	4	38.38	0.01800
CXCL10/IP-10/CRG-2	Increased	8.05	1.96	4	0.00	0.00	4	8.05	0.00380
CXCL11/I-TAC	Decreased	−5.52	17.67	4	474.91	60.96	4	−480.43	0.00026
CXCL9/MIG	Decreased	−82.29	58.26	4	3297.62	1202.06	4	−3379.91	0.01100

Cytokine profiling conducted on *in vitro* co-cultures (4 independent replicates) between human CRC cell lines HCT-116 p53−/− and DLD-1 and TALL-104 revealed 6 cytokines with significantly different treatment effects following 24 h anti-PD-1 treatment (25 μg/mL pembrolizumab).

## DISCUSSION

Several studies have suggested potential mechanisms of HPD/HP which might be complementary or act independently, however, the molecular mechanisms remain elusive [7, 8]. Given the fact that the HPD/HP phenotype is relatively rare with short survival, large cohorts of patients are needed to identify and validate preclinical findings. Thus, mouse models, particularly humanized mouse models, might play a critical role in providing possible mechanisms and biomarkers involved in the process. Although current practice relies on syngeneic mouse models due to their advantages, such as availability and relatively low cost, their use has limitations mainly because of biologic and genetic differences between humans and mice [9]. To overcome some of these limitations, humanized mice engrafted with a partially functioning human immune system have the potential for better predictive evaluation of cancer immunotherapies in preclinical studies. In humanized mouse models, mice engrafted with human CD34+ hematopoietic stem cells have stable multi-lineage engraftment of human immune cell populations without graft-versus-host disease (GVHD), whereas peripheral blood mononuclear cell (PBMC)-engrafted mice have functional T-cell populations but develop GVHD within several weeks following engraftment. Limiting factors to these models include the substantial cost and limited supply of mice.

In our study, we used NCRU-nude athymic mice which lack T-cells or T-cell function and injected human colon carcinoma cell lines subcutaneously into their right flank before injecting TALL-104 intraperitoneally. TALL-104 is a leukemic T-cell line with surface markers typical of CTL’s (CD3+/TCRαβ+, CD8+) and CD56+. TALL-104 is well-known for its MHC non-restricted tumoricidal activity even after IL-2 deprivation and it has the ability to discriminate between tumor and normal cells [10]. Moreover, ^111^In-labeled TALL-104 cells were detected within the primary tumor mass and at the site of distant metastases in tumor-bearing animals [11, 12].

We observed TALL-104 tumoricidal activity both *in vivo* and *in vitro*. TALL-104 cells were detected within the primary tumor mass (Figure 2B). It was previously shown that non-irradiated TALL-104 cells intraperitoneally injected into SCID mice are able to proliferate in the absence of exogenous administration of recombinant human (rh) IL-2 without causing GVHD [13], and this was confirmed in our study. Earlier comparison of the kinetics of biodistribution of irradiated and nonirradiated TALL-104 cells in the peripheral blood, bone marrow (BM), spleen, lung, and liver showed that both were present in all of the organs/tissues examined on day 1 but irradiated TALL-104 cells were only detectable in the BM on day 5. However, non-irradiated TALL-104 cells persisted in every organ [13].

We also showed persistence of TALL-104 cells in the periphery and within tumor masses at the end of the study (Figure 2A and 2B). Since TALL-104 is a leukemic cell line, leukemic effects on mice were previously examined. Lethally irradiated TALL-104 cells were shown to be no longer leukemogenic in SCID mice, whereas non-irradiated TALL-104 (1 × 10^7^ cells) induced leukemia with symptoms of lethargy, enlarged abdomen, respiratory distress, ruffled fur and hunched posture within 10–11 weeks [14]. In our study, we injected much lower numbers of non-irradiated TALL-104 cells (total of 1 × 10^6^ cells/mouse) for a shorter study period (~4 weeks). As expected, we did not observe any GVHD, leukemogenesis, or other concerning side effects.

The FDA granted approvals to pembrolizumab and nivolumab, human IgG4k monoclonal antibodies against PD-1, for different types of cancers at different stages with more potential approvals coming ahead [15]. Since neither pembrolizumab nor nivolumab recognize murine PD-1, surrogate anti-mouse PD-1 antibodies are being used in most murine models [16]. Humanized mouse models, on the other hand, allow for use of human monoclonal antibodies. For instance, significant tumor growth delay following pembrolizumab therapy was shown in the Onco-HuNSG mouse model using allogeneic but HLA partially matched CD34+ human pluripotent stem cell (HPSC) donors and tumors [17]. The same study showed that the efficacy of pembrolizumab was mediated by human CD8+ T cells. Similarly, in another study, treatment with pembrolizumab or nivolumab inhibited tumor growth significantly in humanized mice and correlated with an increased number of CTL’s [18]. In the current study, pembrolizumab in the presence of cytotoxic TALL-104 cells appears to slow down the growth of the HCT-116 p53+/+ cell line (Figure 1B), which was shown to be resistant to T-cell mediated killing both *in vivo* and *in vitro* (Figure 1B and Figure 3B, respectively), however, this effect did not reach statistical significance. Several reasons may underlie the lack of documented significance of this therapeutic effect, including the low number of T-cells (1 × 10^6^) by i.p injection and possible intrinsic/*de novo* resistance of the HCT-116 p53+/+ cell line despite having the MSI-H (MMR-d) phenotype [19]. The current number of animals used in our experiment may have contributed to the current results. Another tested colorectal cancer cell line, DLD-1, appears sensitive to T-cell mediated tumor cell killing both *in vivo* and *in vitro* (Figure 1B and Figure 3B). However, pembrolizumab did not increase T-cell mediated killing of DLD1 either *in vivo* or *in vitro*. This could be partially explained by the lack of basal PD-L1 expression in DLD-1, which shows elevated PD-L1 expression levels in response to pembrolizumab treatment (Figure 3A).

Several recent studies using murine models have suggested possible underlying immunological mechanisms of HPD. Lo Russo et al. [20] suggested a role of innate immunity with tumor-associated macrophages (TAM) in HPD using murine models as well as NSCLC patient tumor samples. Using athymic nude mice implanted with the human H460 NSCLC cell line with anti–PD-1 antibody (mouse) treatment, they observed increased tumor growth as well as intra-tumoral macrophages. Similarly, they observed an HPD-like phenotype and increased CD11b+F4/80high macrophages (murine) in NSCLC PDX302-bearing SCID mice (with EGFR mutation) when treated with nivolumab. Furthermore, tumor growth in these models was enhanced by treatment with anti–PD-1 but not anti–PD-1 F(ab)2 fragments suggesting the effect was due to the Fc fragment.

Kamada et al. [21] showed an enrichment in proliferating regulatory T-cells (T-reg’s) in advanced gastric cancer patients with HPD after nivolumab treatment. By generating mice with T-cell-specific PD-1 deficiency, the study investigated the role of PD-1 in T-reg cells (murine), suggesting that blockade promotes cell cycling of T-reg cells and augments T-reg cell-mediated immune suppression. Another report demonstrated PD-1 blockade accelerated growth of M109 (mouse cell line)-xenograft tumors with increased proliferation [22]. The study suggests blockade of cancer-intrinsic PD-1 leads to increased viability observed with PD-1 blockade. These studies are crucial to help with understanding the mechanism of HPD, however, differences between the human and mouse immune systems necessitates further studies to validate the results.

Given the high value of mice with human immune systems for *in vivo* dissection of human immune responses, we aimed to generate an alternate, faster, simpler and less costly humanized mouse model using athymic mice bearing a human cancer cell line and a human cytotoxic T-cell line. Thus, our study shows a working alternative humanized mouse model for cancer immunotherapy. More importantly, humanized mouse models bearing CRC HCT-116 p53−/− xenografts display the HPD phenotype which was not observed in other tested human CRC cell lines including HCT-116 p53+/+ and DLD-1 (mutant p53/Ser^241^Phe) (Figure 1B). The importance of the tumor suppressor p53 has been established in many studies and a growing literature supports the p53 status of the cancer cell having a potentially profound impact on the immune response [23]. The mechanistic role of loss of p53 in the current HPD humanized model with HCT-116 cell line remains unclear and requires further mechanistic studies, although it may be important in a background of MMR-d and cytokine effects. The HPD/HP phenotype in our model appears to be independent of MDM2 or MDM4/MDMX. However, we did not test whether reduction or blockade of MDM2 or MDM4/MDMX would reverse the HPD/HP phenotype *in vivo*.

Previous murine models with HPD have lacked human mature functioning T-cells. Our HPD model, on the other hand, has a functioning human T-cell line, TALL-104, which shows phenotypic characteristics of CTL’s. It should be noted that TALL-104 has a CD56 surface marker in addition to its typical markers of CTL’s. A subset of human T-lymphocytes expresses the natural killer cell-associated receptor CD56. CD56+ T-cells were shown to have strong immunostimulatory effector functions, including cytokine production such as IFN-γ, IL-4, IL-13 and an efficient cytotoxic capacity [24, 25]. Flow cytometry analysis showed a similar percentage of TALL-104 cells in the periphery and tumor sites between HPD and other tested cell lines with or without pembrolizumab (Figure 2A and 2B).

Human cytokine profiling revealed several cytokines including IFN-γ, TRAIL R2/TNFRSF10B, TRANCE/TNFSF11/RANK L, CCL2/JE/MCP-1, Chitinase 3-like 1, IL-4 and TNF-α (Table 3) with a unique response in the HPD model which might help explain the underlying immune mechanism(s), identify potential biomarker(s) or help guide rational immunotherapy strategies. Among several suggested mechanisms for HPD based on clinical and preclinical studies, potential roles of cytokines have also been suggested [2, 7]. Because cytokines have overlapping functions and act in networks, changes of cytokine levels are difficult to interpret and require further functional studies. IFN-γ was significantly found to be elevated in our HPD model group compared to other groups. Growing evidence supports IFN-γ-dependent mechanisms potentially having an important role in the development of HPD. These include but are not limited to activation of the JAK/STAT signaling pathway resulting in PD-L1 upregulation [26], the recruitment of granulocytic myeloid-derived suppressor cells (MDSCs) to the tumor microenvironment following ICI therapy through the IFN-γ-dependent activation of the inflammasome pathway [27], and immunosuppressive enzyme indoleamine 2,3-dioxygenase (IDO1) induction and depletion of effector T cells by activation-induced cell death [28].

TRAIL-R2/TNFRSF10B and TRANCE/TNFSF11/RANK L were found to have increased levels, whereas CCL2/JE/MCP-1, Chitinase 3-like 1, IL-4 and TNF-α were found to have decreased levels in the HPD model (Table 3). Potential roles of these cytokines in HPD require further investigation and it would be of interest to determine whether therapeutic targeting of TRAIL-R2 or other cytokines might have utility in treating HPD/HP models. Of note, analysis of the immune population in the current model is limited to human immune cells. Thus, further studies dissecting potential roles of murine immune cells contributing to the current HPD model may be warranted.

In conclusion, we report a novel humanized MMR-d colon cancer HPD model which may serve as a tool to facilitate understanding of the pathophysiology of HPD and which may help identify biomarker(s) and novel therapeutic targets or strategies for cancer immunotherapy. Given its relatively low cost, feasibility and relatively simple establishment procedures, the current working humanized HPD/HP mouse model may be useful in studying human CTL’s and immune checkpoint inhibitors in other cancer cell line–derived human xenografts, patient-derived xenografts or patient-derived organoid systems. Future experiments can test patient-derived autologous T-cells, other immune cells or subsets, as well as potential impact of hormones and gender of mice as biological variables. A variety of gain-of-function or loss-of-function screens could be performed to characterize the molecular determinants of HPD/HP *in vivo* and this may uncover additional biomarkers or synergies to anticipate and prevent or treat HPD/HP in patients receiving immune checkpoint blockade therapy.

## MATERIALS AND METHODS

### Generation of humanized xenograft tumors with human T-cell line

We used 8–10-week-old female NCRU-nude athymic mice that were anesthetized with 100 mg/kg ketamine and 10 mg/kg xylazine before 1 × 10^6^ cells of either HCT-116 p53+/+, HCT-116 p53−/−, or DLD-1 suspended in 50 μL ice-cold PBS and 50 μL Corning^®^ Matrigel^®^ Matrix (cat # CB-40234, Fisher Scientific, Waltham, MA, USA) were injected subcutaneously into their right flank. Tumors were allowed to grow for 1-week pre-randomization up to a volume of 100–200 mm^3^ (all measurements done by caliper, with volume calculated as [(short axis)^2^ × (long axis)]/2) before being randomly assigned to one of three treatment groups (vehicle, T-cell, T-cell + Pembrolizumab) such that initial group mean tumor volumes were roughly equal.

### Humanized xenograft tumors with human T-cell line experiment

Following randomization (day 0), 0.75 × 10^6^ TALL-104 CD8+ human cytotoxic T-cells were injected intraperitoneally to each mouse assigned to either the T-cell or T-cell + Pembrolizumab treatment group, suspended in 100 μL ice-cold PBS. Mice assigned to the vehicle group received 100 μL ice-cold PBS by the same administration route without cells. A second T-cell injection was carried out on day 7, with mice in T-cell and T-cell + Pembrolizumab groups receiving 0.25 × 10^6^ TALL-104 CD8+ human T-cells in 100 μL ice-cold PBS and those in vehicle groups receiving 100 μL ice-cold PBS. Mice in the T-cell + Pembrolizumab treatment group received an initial primer dosage of 10 mg/kg Pembrolizumab in a total volume of 100 μL PBS administered intraperitoneally on Day 1, followed by 5 mg/kg Pembrolizumab in a total volume of 100 μL PBS administered intraperitoneally on day 4 and then twice a week beginning on day 8 up until the end of the experiment. Mice in the vehicle or T-cell alone treatment groups received 100 μL plain PBS on all treatment days.

Caliper measurements of tumor volumes were taken twice a week and mouse weights were measured once weekly. The experiment was continued until such time that mouse tumor burden reached the ethically allowed limits (2000 mm^3^ or approximately 10% of body weight). Mice were euthanized and samples collected at the end of the experiment in accordance with Brown University-approved IACUC protocols.

### Immunohistochemistry

Tumor tissue sections were used for immunohistochemical staining against human Ki67. Resected HCT-116 p53+/+ and HCT-116 p53−/− tumors from 3 mice in each group were fixed in formalin for 24–72 hours before being embedded in paraffin and 4 μm sections were cut onto charged glass slides. Slides were deparaffinized with assorted xylenes and then rehydrated with decreasing concentrations of EtOH, before heat-induced epitope retrieval was conducted for 20 minutes using a vegetable steamer to maintain a retrieval buffer (pH 6 sodium citrate solution) temperature of 95 ± 3°C. Slides were immersed in 3% H_2_O_2_ for 10 minutes to quench any endogenous peroxidases, permeabilized with TBST for 10 minutes, and blocked with 2.5% horse serum for 40 minutes before being incubated with primary antibodies (Ki67 5 μg/mL cat # ab15580, Abcam, Cambridge, UK) for 16 hours at 4°C. HRP-conjugated secondary anti-rabbit (ImmPRESS^®^ HRP Horse Anti-Rabbit IgG PLUS Polymer Kit, Peroxidase, cat # MP-7801, Vector Labs, San Francisco, CA, USA) antibodies were then applied to the slides for 40 minutes at 25°C, before development with 3,3′-diaminobenzidine (DAB Substrate Kit, Peroxidase (HRP), with Nickel, (3,3′-diaminobenzidine), cat # SK-4100, Vector Labs, San Francisco, CA, USA) for 5 minutes. Gill’s haematoxylin was applied for 4 seconds as a counterstain before slides were dehydrated with increasing concentrations of EtOH before mounting, application of a cover slip, and imaging.

Slides were imaged on a Nikon Y-THM Multiview Main Teaching Unit microscope using a Diagnostic Instruments, Inc. model 18.2 color mosaic camera paired with SPOT Basic version 5.3.5 software.

Ki67 quantification was conducted using the Fiji distribution of ImageJ. A total of 3-fields from each of 3-stained slides per group bearing HCT-116 p53−/− and p53+/+ xenografted tumors were imaged at 200× and loaded into the program and the DAB stain isolated. Ki67 signal strength was assessed with assistance of software from each of 3 ROI containing fully non-necrotic tissue on each field. An expression per high-power field (HPF) value was obtained for each slide by averaging all 9 ROI values per slide (3 per field, 3 fields per slide), and groups were compared with Student’s *t* test corrected for multiple comparisons by the Bonferroni method.

### Flow cytometry

A total of 4 spleens and 4 tumors from each group with T-cells were processed for flow cytometry analysis. In brief, resected tumors were pre-processed by finely mincing with razor blades before digestion at 37°C for 1 hour in digestion buffer (75 U/mL collagenase IV, 125 μg/mL dispase II, 1% penicillin/streptomycin, in PBS). Digested tumors and spleens were then passed through a 70 μm filter and washed with PBS. Cells were isolated from the resulting suspension by centrifugation and immersion in 2 mL complete RPMI-1640 media (HyClone RPMI 1640 cat # SH30027.02, Cytiva Life Sciences, Marlborough, MA with 10% FBS and 1% penicillin/streptomycin), and immune cells were further isolated by adding 2 mL Ficoll-Paque PLUS (Ficoll-Paque PLUS cat # 17144002, Cytiva Life Sciences, Marlborough, MA, USA) below the cell suspension and centrifuging for 30 minutes at 400× g with minimal acceleration and no deceleration. Cells on the interface layer following centrifugation were collected and washed before being stained for flow analysis.

Flow cytometry viability staining was conducted by suspending murine spleen and tumor single cell suspensions in Zombie Violet solution (BioLegend, San Diego, CA, USA) according to manufacturer instructions for 30 minutes at room temperature. Staining for membrane surface proteins was conducted using conjugated primary antibodies for 1 hour on ice, according to manufacturer instructions. The following antibodies were used for the described experiments: anti-CD45 Monoclonal Antibody (HI30) Alexa Fluor 561 (eBioscience™, San Diego, CA, USA), anti-CD3 Monoclonal Antibody (UCHT1) APC (eBioscience™, San Diego, CA, USA), anti-CD4 Monoclonal Antibody (OKT4 (OKT-4)) Alexa Fluor 488 (eBioscience™, San Diego, CA, USA). Cells were fixed using IC Fixation Buffer (eBioscience™, San Diego, CA, USA) for 30 minutes according to manufacturer instructions. Cells were resuspended in Flow Cytometry Staining Buffer (R&D Systems, Minneapolis, MN, USA) and were analyzed using a BD Biosciences LSR II and FlowJo version 10.1 (FlowJo, Ashland, OR, USA). Gating strategies are as follows: CD8+ T cell: live/CD45+/CD3+/CD4- and live/CD45+/CD3+/CD4−.

### Human cytokine profiling

Human cell line culture supernatants and murine plasma samples were analyzed using an R&D systems Human Premixed Multi-Analyte Kit (R&D Systems, Inc., Minneapolis, MN, USA) and a Luminex 200 Instrument (LX200-XPON-RUO, Luminex Corporation, Austin, TX, USA) according to the manufacturer’s instructions. Sample levels of TNF-alpha, 4-1BB/TNFRSF9/CD137, IL-8/CXCL8, Ferritin, IFN-beta, IL-10, CCL2/JE/MCP-1, VEGF, CXCL13/BLC/BCA-1, IFN-gamma, CCL20/MIP-3 alpha, CCL3/MIP-1 alpha, CCL22/MDC, CCL4/MIP-1 beta, Fas Ligand/TNFSF6, IL-17/IL-17A, IL-2, BAFF/BLyS/TNFSF13B, GM-CSF, CXCL5/ENA-78, TRANCE/TNFSF11/RANK L, CXCL9/MIG, G-CSF, IFN-gamma R1/CD119, VEGFR3/Flt-4, C-Reactive Protein/CRP, CXCL11/I-TAC, IL-21, CXCL14/BRAK, IL-6, Fas/TNFRSF6/CD95, TRAIL R3/TNFRSF10C, IL-4, CCL5/RANTES, PD-L1/B7-H1, CCL7/MCP-3/MARC, Chitinase 3-like 1, CXCL10/IP-10/CRG-2, IL-1 beta/IL-1F2, IL-7, Prolactin, CCL8/MCP-2, TRAIL R2/TNFRSF10B, M-CSF, IL-15, Granzyme B, IFN-alpha, TREM-1, IL-12/IL-23 p40, TRAIL/TNFSF10, CCL11/Eotaxin, and IL-18/IL-1F4. Sample values are reported in pg/mL.

### Cell lines

Cell lines were purchased from ATCC, unless otherwise stated, and maintained in their appropriate growth medium at 37°C and 5% CO2. The human colon adenocarcinoma cell lines HCT116 p53 wild-type and HCT116 p53−/−, obtained from the laboratory of Bert Vogelstein at Johns Hopkins University, were cultured in McCoy’s 5A medium, supplemented with 10% FBS and 2 mmol/l glutamine, 100 U/ml penicillin, and 100 μg/ml streptomycin. Another human colon adenocarcinoma cell line, DLD-1, (mutant p53/Ser^241^Phe) was cultured in RPMI-1640 containing 10% FBS with 2 mmol/l glutamine, 100 U/ml penicillin, and 100 μg/ml streptomycin. The human T-cell line, TALL-104 (expressing CD2 +; CD3 +; CD7 +; CD8 +; CD56 +; CD4 −; CD16 −) was cultured in RPMI-1640 containing 20% FBS and 2 mmol/l glutamine, 100 U/ml penicillin, and 100 μg/ml streptomycin. Recombinant human IL-2 (Miltenyi cat# 130-097744) with a final concentration of 100 units/mL was added to TALL-104 culture media.

### Western blot analysis

Cells were lysed in RIPA buffer (sigma) containing cocktail protease inhibitors (Roche). Equal amounts of cell lysates were electrophoresed through 4–12% SDS-PAGE then transferred to PVDF membranes. The transferred PVDF membranes were blocked with 5% skim milk for 1 hour at room temperature, then incubated with primary antibodies in the blocking buffer at 4°C overnight. Antibody binding was detected on PVDF with appropriate HRP-conjugated secondary antibodies by the syngene imaging system. The following antibodies were used for immunoblotting: anti-p53 (DO-1) and anti-MDM2 (SMP14) from Santa Cruz; anti-PD-L1 (E1L3N), Anti-MDMX/MDM4 (ab16058) from Abcam; anti-PD1 (D4W2J) from Cell Signaling; anti-P21 (Ab-1) from EMD Millipore and anti-Ran from BD bioscience.

### T-cell co-culture system and microscopic imaging for data analysis

CellTracker™ CMFDA (5-chloromethylfluorescein diacetate) green fluorescent dye (5 μM) was used to stain and detect living tumor cells or T-cells as described before [29]. Before co-culture with T-cells, culture media was removed and pre-warmed CellTracker™ in working solution was added as instructed in manufacturer’s protocol (Invitrogen, Waltham, MA, USA). Working solution with the CellTracker™ was replaced with fresh media after 30 minutes of incubation at 37°C. Green fluorescent tumor cells were co-cultured with or without 25 μg/mL pembrolizumab pretreated cytotoxic T-cells (TALL-104) with 1:1 effector to target cell ratio (E:T) for 20 hours. RPMI-1640 media containing 20% FBS and 100 units/mL IL-2 was used in the co-culture system. 1 μM red fluorescent ethidium homodimer-1 (EthD-1) was added and incubated for another 30 minutes to detect dead cells (Invitrogen, Waltham, MA, USA). For the quantification of dead/total cells, fluorescence microscopy was used to take images at 10× magnification. The number of red/green color cells in random fields was counted by 2 independent investigators and expressed as a dead/live cell ratio. At least 100 cells were evaluated per sample, with 3 independent replicates.

### Statistical analysis

For *in vivo* studies; statistical differences between treatment groups within a cell line were determined using a one-way ANOVA F-test with a minimum significance level of *P* < 0.05. Groups were further analyzed with Student’s *t* testing, as supported by *F*-tests for variance testing, and Bonferroni correction for multiple comparisons with a family-wise error rate set to 0.05, unless otherwise specified. The following symbols ^*^, ^**^, and ^***^ were coded to signify *P* < 0.05, *P* < 0.01, and *P* < 0.001, respectively. Statistical analysis and generation of charts in figures related to *in vivo* studies was carried out using the R Project for Statistical Computing.

For *in vitro* studies; the statistical significance of differences between groups was determined using the Student’s *t* test unless otherwise specified. The minimal level of significance was *P* < 0.05. Following symbols ^*^ and ^**^ represent, *P* < 0.05 and *P* < 0.01, respectively.

## SUPPLEMENTARY MATERIALS


