# Supplementary material for: Hyperprogression of a mismatch repair-deficient colon cancer in a humanized mouse model following administration of immune checkpoint inhibitor pembrolizumab
Source: Oncotarget. 2021 Oct 12;12(21):2131–46. doi: 10.18632/oncotarget.28086 (PMC8522841; doi:10.18632/oncotarget.28086)
Supplement: Supplementary file 1 [file oncotarget-12-2131-s001.pdf]

Hyperprogression of a mismatch repair-deficient colon cancer in a humanized mouse model following administration of immune checkpoint inhibitor pembrolizumab

SUPPLEMENTARY MATERIALS

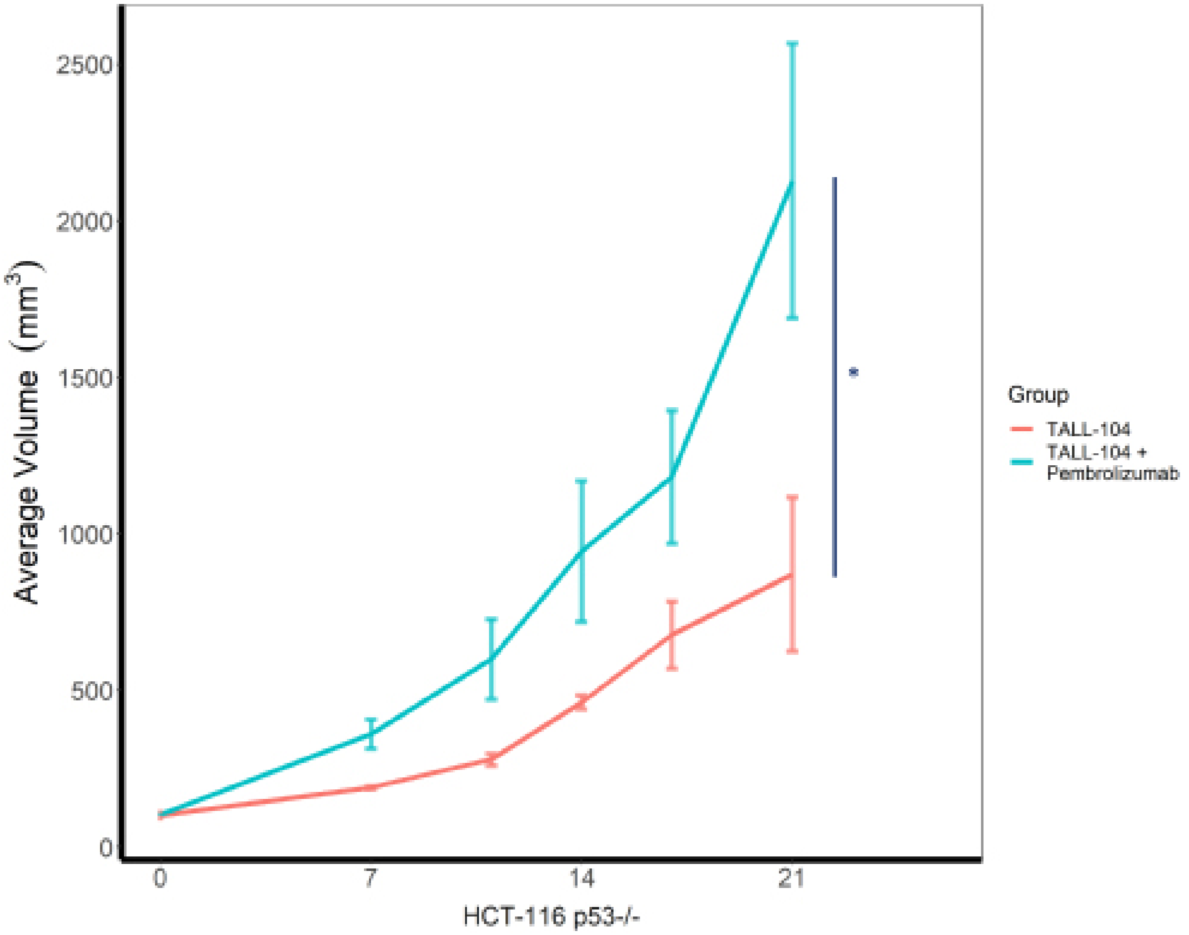

Supplementary Figure 1: Independent experiment to reproduce HPD/HP phenotype in the humanized TALL-104 HCT-116 p53−/− model. Tumor growth curve of selected humanized control and pembrolizumab treatment group conditions show the HPD/HP phenotype. For each group; $n = 4$, significance ($P < 0.05$).
